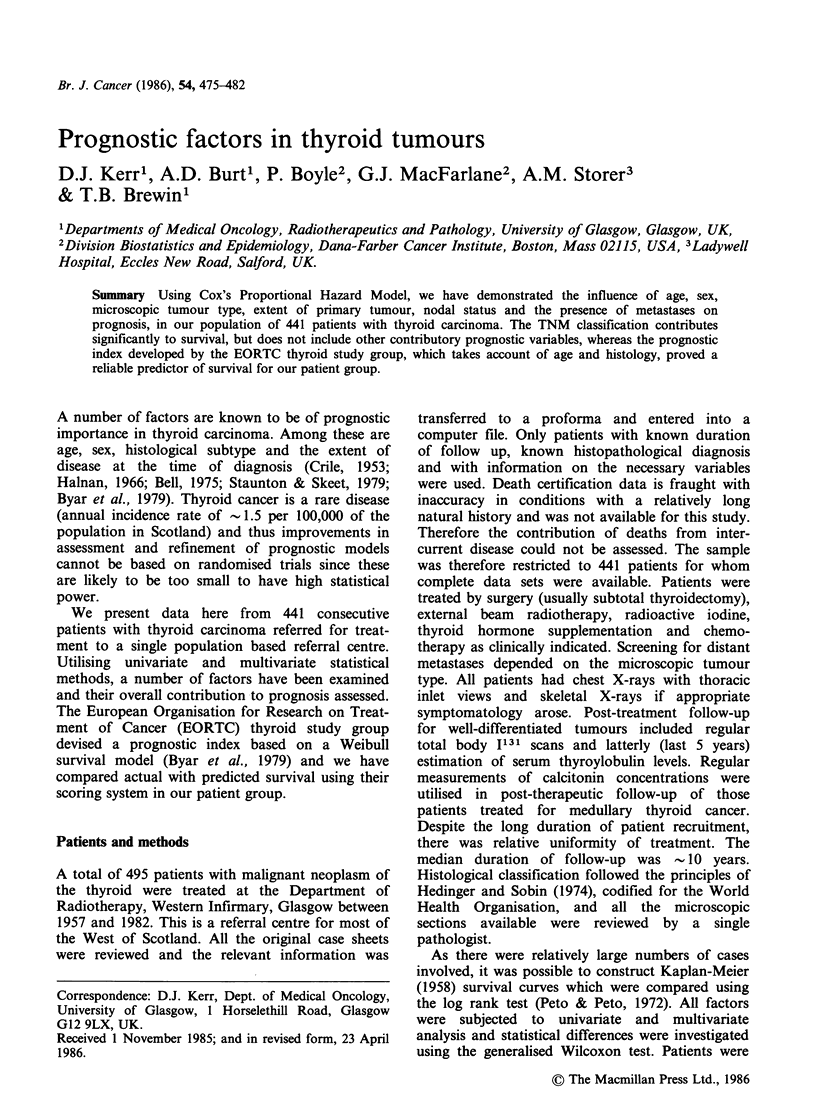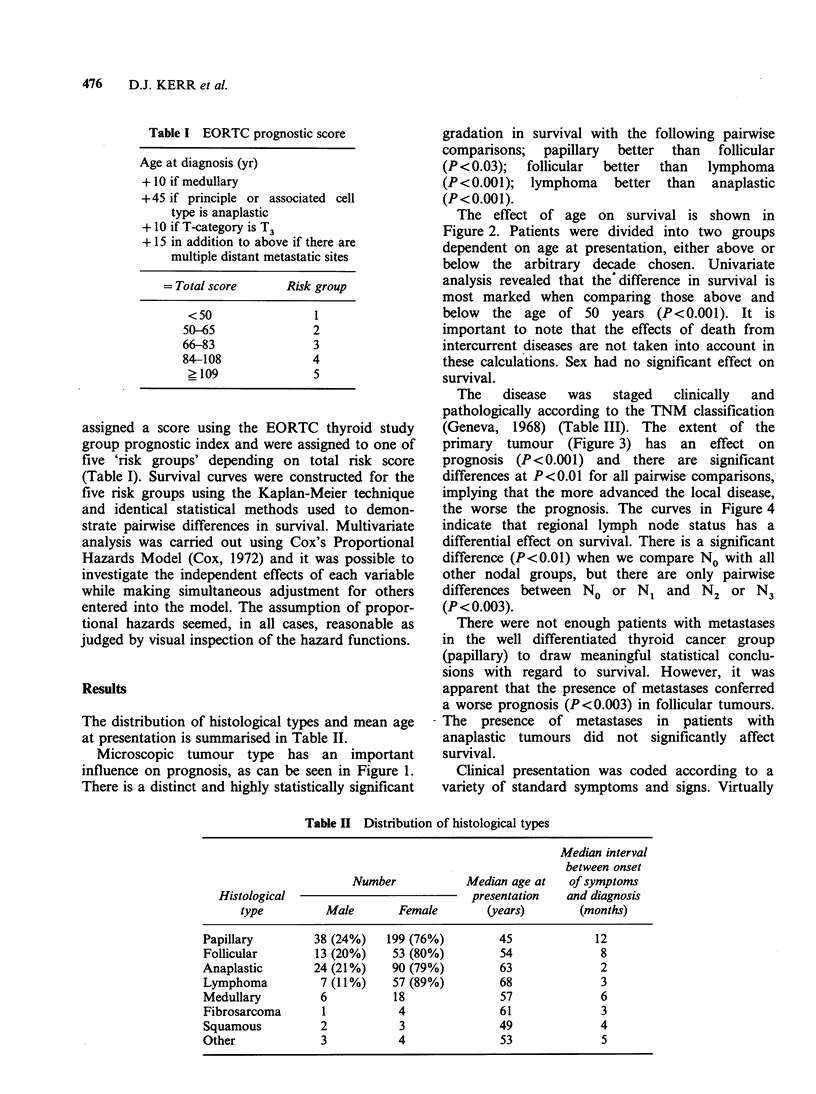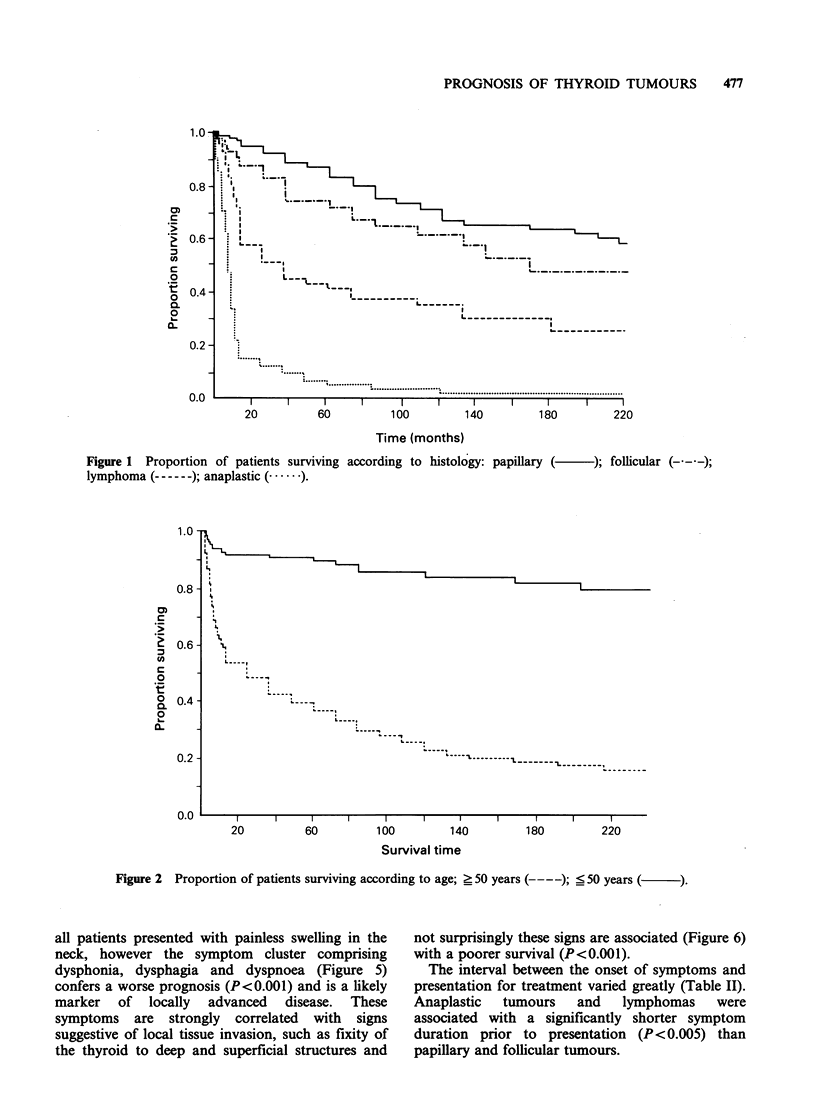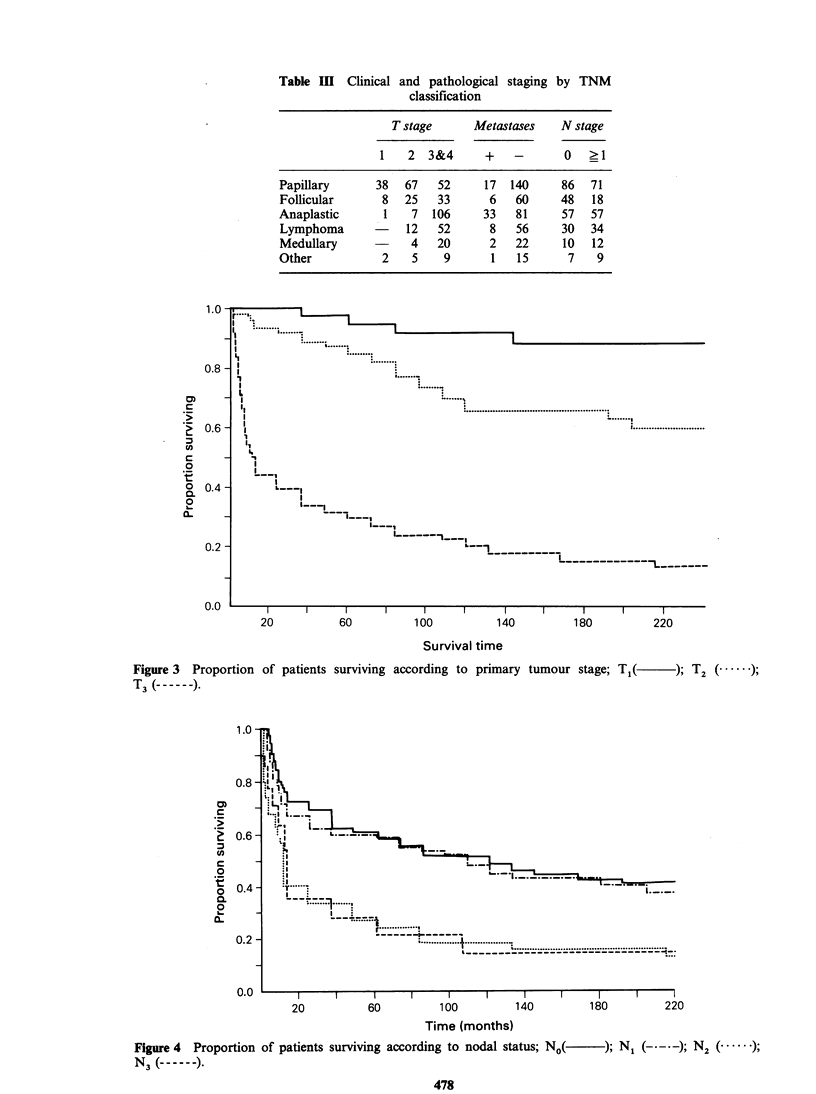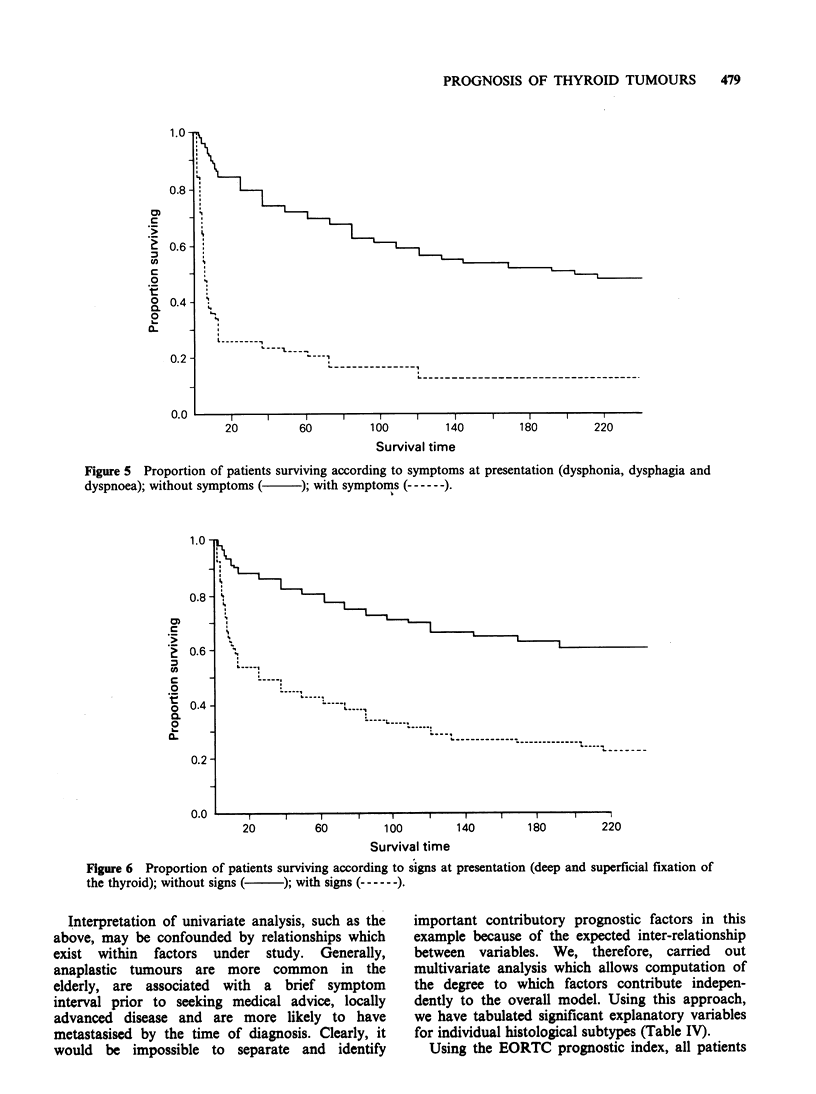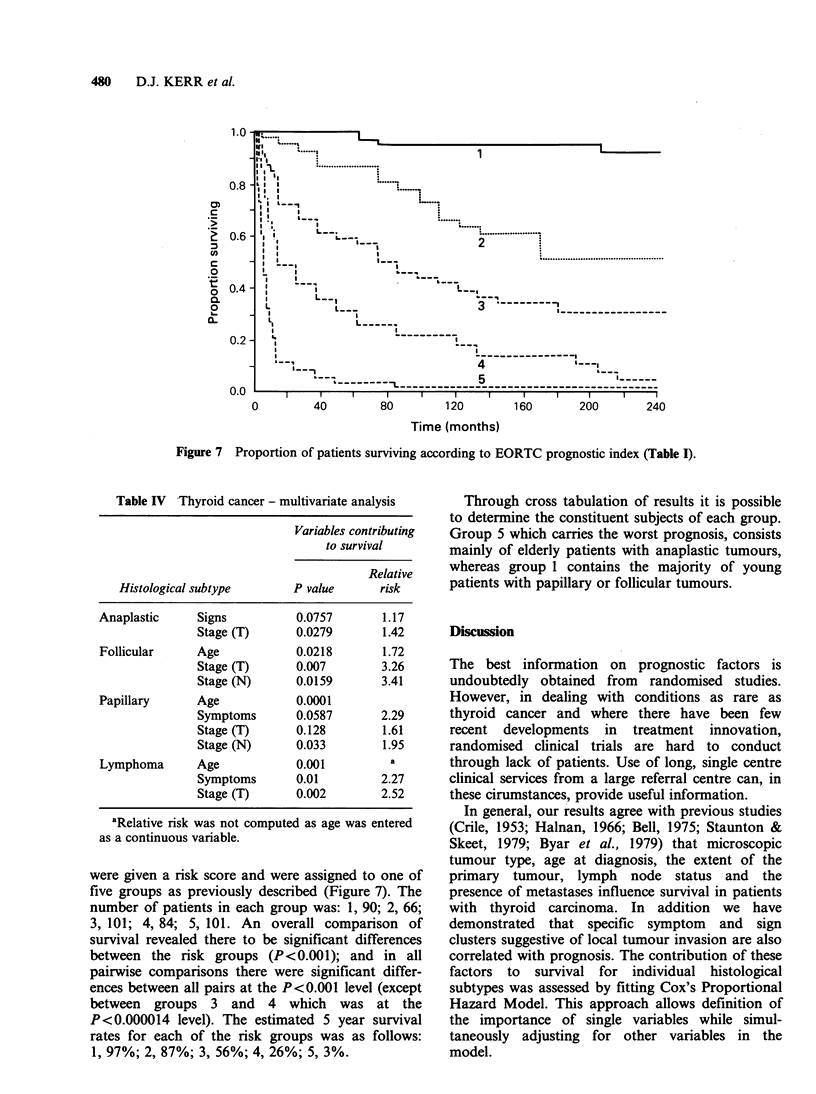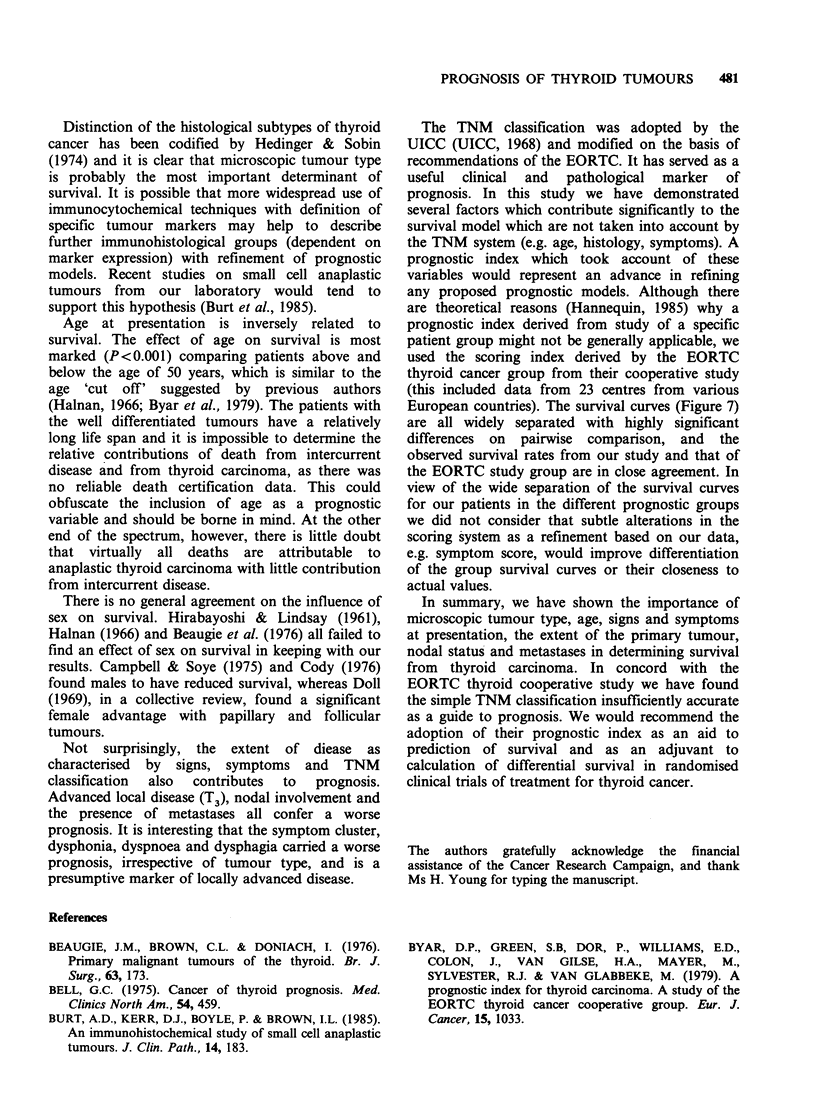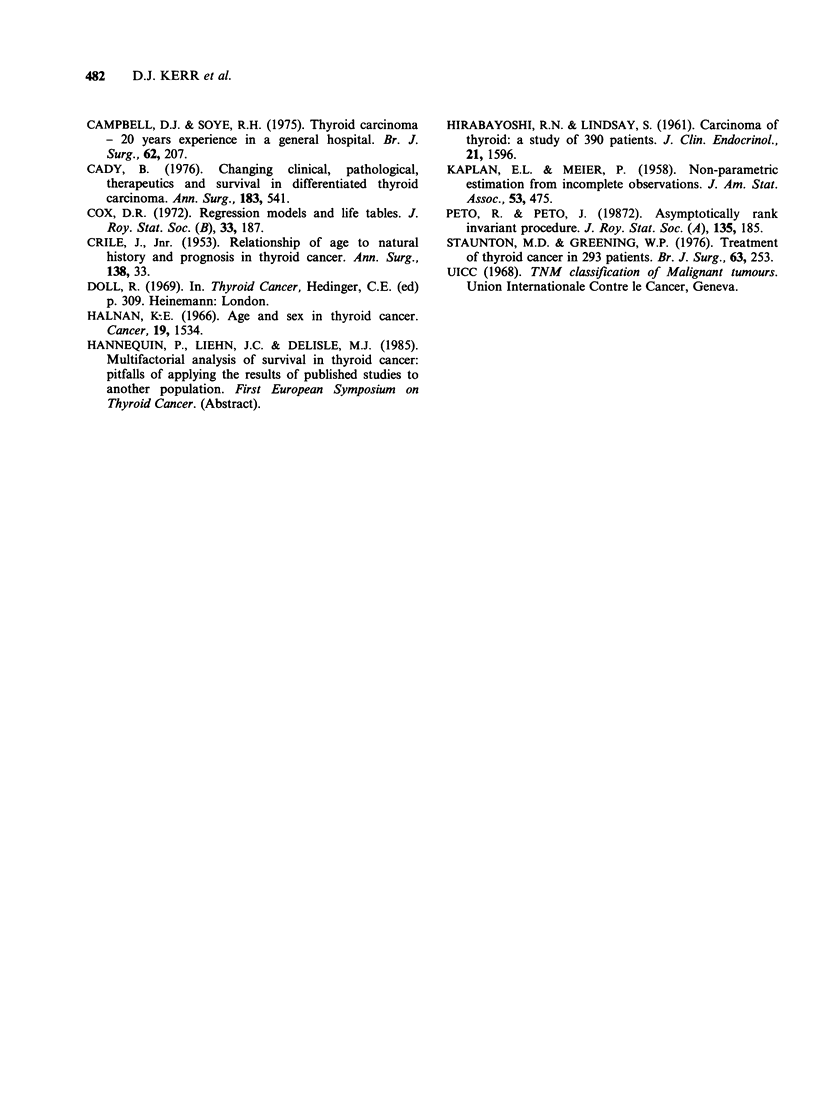# Prognostic factors in thyroid tumours.

**DOI:** 10.1038/bjc.1986.200

**Published:** 1986-09

**Authors:** D. J. Kerr, A. D. Burt, P. Boyle, G. J. MacFarlane, A. M. Storer, T. B. Brewin

## Abstract

Using Cox's Proportional Hazard Model, we have demonstrated the influence of age, sex, microscopic tumour type, extent of primary tumour, nodal status and the presence of metastases on prognosis, in our population of 441 patients with thyroid carcinoma. The TNM classification contributes significantly to survival, but does not include other contributory prognostic variables, whereas the prognostic index developed by the EORTC thyroid study group, which takes account of age and histology, proved a reliable predictor of survival for our patient group.


Br. J. Cancer (1986), **54**, 475–482

### Prognostic factors in thyroid tumours

D.J. Kerr[1], A.D. Burt[1], P. Boyle[2], G.J. MacFarlane[2], A.M. Storer[3] & T.B. Brewin[1]

[1]*Departments of Medical Oncology, Radiotherapeutics and Pathology, University of Glasgow, Glasgow, UK,* [2]*Division Biostatistics and Epidemiology, Dana-Farber Cancer Institute, Boston, Mass 02115, USA,* [3]*Ladywell Hospital, Eccles New Road, Salford, UK.*

**Summary** Using Cox's Proportional Hazard Model, we have demonstrated the influence of age, sex, microscopic tumour type, extent of primary tumour, nodal status and the presence of metastases on prognosis, in our population of 441 patients with thyroid carcinoma. The TNM classification contributes significantly to survival, but does not include other contributory prognostic variables, whereas the prognostic index developed by the EORTC thyroid study group, which takes account of age and histology, proved a reliable predictor of survival for our patient group.

A number of factors are known to be of prognostic importance in thyroid carcinoma. Among these are age, sex, histological subtype and the extent of disease at the time of diagnosis (Crile, 1953; Halnan, 1966; Bell, 1975; Staunton & Skeet, 1979; Byar *et al.*, 1979). Thyroid cancer is a rare disease (annual incidence rate of ~1.5 per 100,000 of the population in Scotland) and thus improvements in assessment and refinement of prognostic models cannot be based on randomised trials since these are likely to be too small to have high statistical power.

We present data here from 441 consecutive patients with thyroid carcinoma referred for treatment to a single population based referral centre. Utilising univariate and multivariate statistical methods, a number of factors have been examined and their overall contribution to prognosis assessed. The European Organisation for Research on Treatment of Cancer (EORTC) thyroid study group devised a prognostic index based on a Weibull survival model (Byar *et al.*, 1979) and we have compared actual with predicted survival using their scoring system in our patient group.

### Patients and methods

A total of 495 patients with malignant neoplasm of the thyroid were treated at the Department of Radiotherapy, Western Infirmary, Glasgow between 1957 and 1982. This is a referral centre for most of the West of Scotland. All the original case sheets were reviewed and the relevant information was transferred to a proforma and entered into a computer file. Only patients with known duration of follow up, known histopathological diagnosis and with information on the necessary variables were used. Death certification data is fraught with inaccuracy in conditions with a relatively long natural history and was not available for this study. Therefore the contribution of deaths from intercurrent disease could not be assessed. The sample was therefore restricted to 441 patients for whom complete data sets were available. Patients were treated by surgery (usually subtotal thyroidectomy), external beam radiotherapy, radioactive iodine, thyroid hormone supplementation and chemotherapy as clinically indicated. Screening for distant metastases depended on the microscopic tumour type. All patients had chest X-rays with thoracic inlet views and skeletal X-rays if appropriate symptomatology arose. Post-treatment follow-up for well-differentiated tumours included regular total body $I^{131}$ scans and latterly (last 5 years) estimation of serum thyroylobulin levels. Regular measurements of calcitonin concentrations were utilised in post-therapeutic follow-up of those patients treated for medullary thyroid cancer. Despite the long duration of patient recruitment, there was relative uniformity of treatment. The median duration of follow-up was ~10 years. Histological classification followed the principles of Hedinger and Sobin (1974), codified for the World Health Organisation, and all the microscopic sections available were reviewed by a single pathologist.

As there were relatively large numbers of cases involved, it was possible to construct Kaplan-Meier (1958) survival curves which were compared using the log rank test (Peto & Peto, 1972). All factors were subjected to univariate and multivariate analysis and statistical differences were investigated using the generalised Wilcoxon test. Patients were

Correspondence: D.J. Kerr, Dept. of Medical Oncology, University of Glasgow, 1 Horselethill Road, Glasgow G12 9LX, UK.
Received 1 November 1985; and in revised form, 23 April 1986.

© The Macmillan Press Ltd., 1986

**Table I** EORTC prognostic score

| Age at diagnosis (yr) | |
|---|---|
| +10 if medullary | |
| +45 if principle or associated cell type is anaplastic | |
| +10 if T-category is $T_3$ | |
| +15 in addition to above if there are multiple distant metastatic sites | |
| *= Total score* | *Risk group* |
| <50 | 1 |
| 50–65 | 2 |
| 66–83 | 3 |
| 84–108 | 4 |
| ≧109 | 5 |

assigned a score using the EORTC thyroid study group prognostic index and were assigned to one of five 'risk groups' depending on total risk score (Table I). Survival curves were constructed for the five risk groups using the Kaplan-Meier technique and identical statistical methods used to demonstrate pairwise differences in survival. Multivariate analysis was carried out using Cox's Proportional Hazards Model (Cox, 1972) and it was possible to investigate the independent effects of each variable while making simultaneous adjustment for others entered into the model. The assumption of proportional hazards seemed, in all cases, reasonable as judged by visual inspection of the hazard functions.

### Results

The distribution of histological types and mean age at presentation is summarised in Table II.

Microscopic tumour type has an important influence on prognosis, as can be seen in Figure 1. There is a distinct and highly statistically significant gradation in survival with the following pairwise comparisons; papillary better than follicular ($P<0.03$); follicular better than lymphoma ($P<0.001$); lymphoma better than anaplastic ($P<0.001$).

The effect of age on survival is shown in Figure 2. Patients were divided into two groups dependent on age at presentation, either above or below the arbitrary decade chosen. Univariate analysis revealed that the difference in survival is most marked when comparing those above and below the age of 50 years ($P<0.001$). It is important to note that the effects of death from intercurrent diseases are not taken into account in these calculations. Sex had no significant effect on survival.

The disease was staged clinically and pathologically according to the TNM classification (Geneva, 1968) (Table III). The extent of the primary tumour (Figure 3) has an effect on prognosis ($P<0.001$) and there are significant differences at $P<0.01$ for all pairwise comparisons, implying that the more advanced the local disease, the worse the prognosis. The curves in Figure 4 indicate that regional lymph node status has a differential effect on survival. There is a significant difference ($P<0.01$) when we compare $N_0$ with all other nodal groups, but there are only pairwise differences between $N_0$ or $N_1$ and $N_2$ or $N_3$ ($P<0.003$).

There were not enough patients with metastases in the well differentiated thyroid cancer group (papillary) to draw meaningful statistical conclusions with regard to survival. However, it was apparent that the presence of metastases conferred a worse prognosis ($P<0.003$) in follicular tumours. The presence of metastases in patients with anaplastic tumours did not significantly affect survival.

Clinical presentation was coded according to a variety of standard symptoms and signs. Virtually

**Table II** Distribution of histological types

| *Histological type* | *Number* | | *Median age at presentation (years)* | *Median interval between onset of symptoms and diagnosis (months)* |
|---|---|---|---|---|
| | *Male* | *Female* | | |
| Papillary | 38 (24%) | 199 (76%) | 45 | 12 |
| Follicular | 13 (20%) | 53 (80%) | 54 | 8 |
| Anaplastic | 24 (21%) | 90 (79%) | 63 | 2 |
| Lymphoma | 7 (11%) | 57 (89%) | 68 | 3 |
| Medullary | 6 | 18 | 57 | 6 |
| Fibrosarcoma | 1 | 4 | 61 | 3 |
| Squamous | 2 | 3 | 49 | 4 |
| Other | 3 | 4 | 53 | 5 |

**Figure 1** Proportion of patients surviving according to histology: papillary (———); follicular (–·–·–); lymphoma (- - - - -); anaplastic (· · · · ·).

**Figure 2** Proportion of patients surviving according to age; ≧50 years (– – – –); ≦50 years (———).

all patients presented with painless swelling in the neck, however the symptom cluster comprising dysphonia, dysphagia and dyspnoea (Figure 5) confers a worse prognosis ($P<0.001$) and is a likely marker of locally advanced disease. These symptoms are strongly correlated with signs suggestive of local tissue invasion, such as fixity of the thyroid to deep and superficial structures and not surprisingly these signs are associated (Figure 6) with a poorer survival ($P<0.001$).

The interval between the onset of symptoms and presentation for treatment varied greatly (Table II). Anaplastic tumours and lymphomas were associated with a significantly shorter symptom duration prior to presentation ($P<0.005$) than papillary and follicular tumours.

**Table III** Clinical and pathological staging by TNM classification

| | *T stage* | | | *Metastases* | | *N stage* | |
|---|---|---|---|---|---|---|---|
| | 1 | 2 | 3&4 | + | − | 0 | ≥1 |
| Papillary | 38 | 67 | 52 | 17 | 140 | 86 | 71 |
| Follicular | 8 | 25 | 33 | 6 | 60 | 48 | 18 |
| Anaplastic | 1 | 7 | 106 | 33 | 81 | 57 | 57 |
| Lymphoma | — | 12 | 52 | 8 | 56 | 30 | 34 |
| Medullary | — | 4 | 20 | 2 | 22 | 10 | 12 |
| Other | 2 | 5 | 9 | 1 | 15 | 7 | 9 |

**Figure 3** Proportion of patients surviving according to primary tumour stage; $T_1$(———); $T_2$ (······); $T_3$ (------).

**Figure 4** Proportion of patients surviving according to nodal status; $N_0$(———); $N_1$ (–·–·–); $N_2$ (······); $N_3$ (------).

**Figure 5** Proportion of patients surviving according to symptoms at presentation (dysphonia, dysphagia and dyspnoea); without symptoms (———); with symptoms (- - - - - -).

**Figure 6** Proportion of patients surviving according to signs at presentation (deep and superficial fixation of the thyroid); without signs (———); with signs (- - - - - -).

Interpretation of univariate analysis, such as the above, may be confounded by relationships which exist within factors under study. Generally, anaplastic tumours are more common in the elderly, are associated with a brief symptom interval prior to seeking medical advice, locally advanced disease and are more likely to have metastasised by the time of diagnosis. Clearly, it would be impossible to separate and identify important contributory prognostic factors in this example because of the expected inter-relationship between variables. We, therefore, carried out multivariate analysis which allows computation of the degree to which factors contribute independently to the overall model. Using this approach, we have tabulated significant explanatory variables for individual histological subtypes (Table IV).

Using the EORTC prognostic index, all patients

**Figure 7** Proportion of patients surviving according to EORTC prognostic index (**Table I**).

**Table IV** Thyroid cancer – multivariate analysis

| | | *Variables contributing to survival* | |
|---|---|---|---|
| *Histological subtype* | | *P value* | *Relative risk* |
| Anaplastic | Signs | 0.0757 | 1.17 |
| | Stage (T) | 0.0279 | 1.42 |
| Follicular | Age | 0.0218 | 1.72 |
| | Stage (T) | 0.007 | 3.26 |
| | Stage (N) | 0.0159 | 3.41 |
| Papillary | Age | 0.0001 | |
| | Symptoms | 0.0587 | 2.29 |
| | Stage (T) | 0.128 | 1.61 |
| | Stage (N) | 0.033 | 1.95 |
| Lymphoma | Age | 0.001 | [a] |
| | Symptoms | 0.01 | 2.27 |
| | Stage (T) | 0.002 | 2.52 |

[a]Relative risk was not computed as age was entered as a continuous variable.

were given a risk score and were assigned to one of five groups as previously described (Figure 7). The number of patients in each group was: 1, 90; 2, 66; 3, 101; 4, 84; 5, 101. An overall comparison of survival revealed there to be significant differences between the risk groups ($P<0.001$); and in all pairwise comparisons there were significant differences between all pairs at the $P<0.001$ level (except between groups 3 and 4 which was at the $P<0.000014$ level). The estimated 5 year survival rates for each of the risk groups was as follows: 1, 97%; 2, 87%; 3, 56%; 4, 26%; 5, 3%.

Through cross tabulation of results it is possible to determine the constituent subjects of each group. Group 5 which carries the worst prognosis, consists mainly of elderly patients with anaplastic tumours, whereas group 1 contains the majority of young patients with papillary or follicular tumours.

### Discussion

The best information on prognostic factors is undoubtedly obtained from randomised studies. However, in dealing with conditions as rare as thyroid cancer and where there have been few recent developments in treatment innovation, randomised clinical trials are hard to conduct through lack of patients. Use of long, single centre clinical services from a large referral centre can, in these cirumstances, provide useful information.

In general, our results agree with previous studies (Crile, 1953; Halnan, 1966; Bell, 1975; Staunton & Skeet, 1979; Byar *et al.*, 1979) that microscopic tumour type, age at diagnosis, the extent of the primary tumour, lymph node status and the presence of metastases influence survival in patients with thyroid carcinoma. In addition we have demonstrated that specific symptom and sign clusters suggestive of local tumour invasion are also correlated with prognosis. The contribution of these factors to survival for individual histological subtypes was assessed by fitting Cox's Proportional Hazard Model. This approach allows definition of the importance of single variables while simultaneously adjusting for other variables in the model.

Distinction of the histological subtypes of thyroid cancer has been codified by Hedinger & Sobin (1974) and it is clear that microscopic tumour type is probably the most important determinant of survival. It is possible that more widespread use of immunocytochemical techniques with definition of specific tumour markers may help to describe further immunohistological groups (dependent on marker expression) with refinement of prognostic models. Recent studies on small cell anaplastic tumours from our laboratory would tend to support this hypothesis (Burt *et al.*, 1985).

Age at presentation is inversely related to survival. The effect of age on survival is most marked ($P<0.001$) comparing patients above and below the age of 50 years, which is similar to the age 'cut off' suggested by previous authors (Halnan, 1966; Byar *et al.*, 1979). The patients with the well differentiated tumours have a relatively long life span and it is impossible to determine the relative contributions of death from intercurrent disease and from thyroid carcinoma, as there was no reliable death certification data. This could obfuscate the inclusion of age as a prognostic variable and should be borne in mind. At the other end of the spectrum, however, there is little doubt that virtually all deaths are attributable to anaplastic thyroid carcinoma with little contribution from intercurrent disease.

There is no general agreement on the influence of sex on survival. Hirabayoshi & Lindsay (1961), Halnan (1966) and Beaugie *et al.* (1976) all failed to find an effect of sex on survival in keeping with our results. Campbell & Soye (1975) and Cody (1976) found males to have reduced survival, whereas Doll (1969), in a collective review, found a significant female advantage with papillary and follicular tumours.

Not surprisingly, the extent of diease as characterised by signs, symptoms and TNM classification also contributes to prognosis. Advanced local disease ($T_3$), nodal involvement and the presence of metastases all confer a worse prognosis. It is interesting that the symptom cluster, dysphonia, dyspnoea and dysphagia carried a worse prognosis, irrespective of tumour type, and is a presumptive marker of locally advanced disease.

The TNM classification was adopted by the UICC (UICC, 1968) and modified on the basis of recommendations of the EORTC. It has served as a useful clinical and pathological marker of prognosis. In this study we have demonstrated several factors which contribute significantly to the survival model which are not taken into account by the TNM system (e.g. age, histology, symptoms). A prognostic index which took account of these variables would represent an advance in refining any proposed prognostic models. Although there are theoretical reasons (Hannequin, 1985) why a prognostic index derived from study of a specific patient group might not be generally applicable, we used the scoring index derived by the EORTC thyroid cancer group from their cooperative study (this included data from 23 centres from various European countries). The survival curves (Figure 7) are all widely separated with highly significant differences on pairwise comparison, and the observed survival rates from our study and that of the EORTC study group are in close agreement. In view of the wide separation of the survival curves for our patients in the different prognostic groups we did not consider that subtle alterations in the scoring system as a refinement based on our data, e.g. symptom score, would improve differentiation of the group survival curves or their closeness to actual values.

In summary, we have shown the importance of microscopic tumour type, age, signs and symptoms at presentation, the extent of the primary tumour, nodal status and metastases in determining survival from thyroid carcinoma. In concord with the EORTC thyroid cooperative study we have found the simple TNM classification insufficiently accurate as a guide to prognosis. We would recommend the adoption of their prognostic index as an aid to prediction of survival and as an adjuvant to calculation of differential survival in randomised clinical trials of treatment for thyroid cancer.

The authors gratefully acknowledge the financial assistance of the Cancer Research Campaign, and thank Ms H. Young for typing the manuscript.

**References**

BEAUGIE, J.M., BROWN, C.L. & DONIACH, I. (1976). Primary malignant tumours of the thyroid. *Br. J. Surg.*, **63**, 173.

BELL, G.C. (1975). Cancer of thyroid prognosis. *Med. Clinics North Am.*, **54**, 459.

BURT, A.D., KERR, D.J., BOYLE, P. & BROWN, I.L. (1985). An immunohistochemical study of small cell anaplastic tumours. *J. Clin. Path.*, **14**, 183.

BYAR, D.P., GREEN, S.B, DOR, P., WILLIAMS, E.D., COLON, J., VAN GILSE, H.A., MAYER, M., SYLVESTER, R.J. & VAN GLABBEKE, M. (1979). A prognostic index for thyroid carcinoma. A study of the EORTC thyroid cancer cooperative group. *Eur. J. Cancer*, **15**, 1033.

CAMPBELL, D.J. & SOYE, R.H. (1975). Thyroid carcinoma – 20 years experience in a general hospital. *Br. J. Surg.*, **62**, 207.
CADY, B. (1976). Changing clinical, pathological, therapeutics and survival in differentiated thyroid carcinoma. *Ann. Surg.*, **183**, 541.
COX, D.R. (1972). Regression models and life tables. *J. Roy. Stat. Soc.* (*B*), **33**, 187.
CRILE, J., Jnr. (1953). Relationship of age to natural history and prognosis in thyroid cancer. *Ann. Surg.*, **138**, 33.
DOLL, R. (1969). In. *Thyroid Cancer*, Hedinger, C.E. (ed) p. 309. Heinemann: London.
HALNAN, K.E. (1966). Age and sex in thyroid cancer. *Cancer*, **19**, 1534.
HANNEQUIN, P., LIEHN, J.C. & DELISLE, M.J. (1985). Multifactorial analysis of survival in thyroid cancer: pitfalls of applying the results of published studies to another population. *First European Symposium on Thyroid Cancer*. (Abstract).
HIRABAYOSHI, R.N. & LINDSAY, S. (1961). Carcinoma of thyroid: a study of 390 patients. *J. Clin. Endocrinol.*, **21**, 1596.
KAPLAN, E.L. & MEIER, P. (1958). Non-parametric estimation from incomplete observations. *J. Am. Stat. Assoc.*, **53**, 475.
PETO, R. & PETO, J. (19872). Asymptotically rank invariant procedure. *J. Roy. Stat. Soc.* (*A*), **135**, 185.
STAUNTON, M.D. & GREENING, W.P. (1976). Treatment of thyroid cancer in 293 patients. *Br. J. Surg.*, **63**, 253.
UICC (1968). *TNM classification of Malignant tumours*. Union Internationale Contre le Cancer, Geneva.